# Functional variants of human papillomavirus type 16 demonstrate host genome integration and transcriptional alterations corresponding to their unique cancer epidemiology

**DOI:** 10.1186/s12864-016-3203-3

**Published:** 2016-11-02

**Authors:** Robert Jackson, Bruce A. Rosa, Sonia Lameiras, Sean Cuninghame, Josee Bernard, Wely B. Floriano, Paul F. Lambert, Alain Nicolas, Ingeborg Zehbe

**Affiliations:** 1 Probe Development and Biomarker Exploration, Thunder Bay Regional Research Institute, Thunder Bay, Ontario Canada; 2 Biotechnology Program, Lakehead University, Thunder Bay, Ontario Canada; 3 McDonnell Genome Institute, Washington University School of Medicine, St. Louis, MO USA; 4 NGS platform, Institut Curie, PSL Research University, 26 rue d’Ulm, 75248 Paris, Cedex France; 5 Northern Ontario School of Medicine, Lakehead University, Thunder Bay, Ontario Canada; 6 Department of Biology, Lakehead University, Thunder Bay, Ontario Canada; 7 Department of Chemistry, Lakehead University, Thunder Bay, Ontario Canada; 8 McArdle Laboratory for Cancer Research, University of Wisconsin School of Medicine and Public Health, Madison, WI USA; 9 Institut Curie, PSL Research University, Centre National de la Recherche Scientifique UMR3244, Sorbonne Universités, Paris, France

**Keywords:** Human papillomavirus, HPV16, E6 oncogene variants, Organotypic rafts, Viral integration, Transcriptomics, Pathogen-host relationship

## Abstract

**Background:**

Human papillomaviruses (HPVs) are a worldwide burden as they are a widespread group of tumour viruses in humans. Having a tropism for mucosal tissues, high-risk HPVs are detected in nearly all cervical cancers. HPV16 is the most common high-risk type but not all women infected with high-risk HPV develop a malignant tumour. Likely relevant, HPV genomes are polymorphic and some HPV16 single nucleotide polymorphisms (SNPs) are under evolutionary constraint instigating variable oncogenicity and immunogenicity in the infected host.

**Results:**

To investigate the tumourigenicity of two common HPV16 variants, we used our recently developed, three-dimensional organotypic model reminiscent of the natural HPV infectious cycle and conducted various “omics” and bioinformatics approaches. Based on epidemiological studies we chose to examine the HPV16 Asian-American (AA) and HPV16 European Prototype (EP) variants. They differ by three non-synonymous SNPs in the transforming and virus-encoded E6 oncogene where AAE6 is classified as a high- and EPE6 as a low-risk variant. Remarkably, the high-risk AAE6 variant genome integrated into the host DNA, while the low-risk EPE6 variant genome remained episomal as evidenced by highly sensitive Capt-HPV sequencing. RNA-seq experiments showed that the truncated form of AAE6, integrated in chromosome 5q32, produced a local gene over-expression and a large variety of viral-human fusion transcripts, including long distance spliced transcripts. In addition, differential enrichment of host cell pathways was observed between both HPV16 E6 variant-containing epithelia. Finally, in the high-risk variant, we detected a molecular signature of host chromosomal instability, a common property of cancer cells.

**Conclusions:**

We show how naturally occurring SNPs in the HPV16 E6 oncogene cause significant changes in the outcome of HPV infections and subsequent viral and host transcriptome alterations prone to drive carcinogenesis. Host genome instability is closely linked to viral integration into the host genome of HPV-infected cells, which is a key phenomenon for malignant cellular transformation and the reason for uncontrolled E6 oncogene expression. In particular, the finding of variant-specific integration potential represents a new paradigm in HPV variant biology.

**Electronic supplementary material:**

The online version of this article (doi:10.1186/s12864-016-3203-3) contains supplementary material, which is available to authorized users.

## Background

Approximately 20 % of human cancers are caused by infectious agents [1], including >500,000 patients diagnosed annually with human papillomavirus (HPV) associated cancers. Oncogenic HPV, denoted as “high-risk”, is the primary risk factor for cervical cancer due to its exclusive tropism for mucosal tissues [2, 3]. Upon persistent infections of the cervical mucosa, oncogenic HPVs can cause progression from low- to high-grade cervical intraepithelial neoplasias that, without ablative treatment, may develop into invasive carcinomas. At the molecular level HPV is a double-stranded DNA virus and, to date, the sequences of over 200 types have been described [4]. The ~8 kbp genome of HPV contains 8 functional open reading frames (ORFs) that encode 5 early gene products (E1, E2, E5, E6 and E7) and 3 late gene products (E4, L1 and L2). While E1 and E2 are involved in DNA replication and transcriptional regulation of the viral genome [5], HPV’s potent tumourigenicity is primarily due to E6 [6], E7 [7], and E5 [8]. L1 and L2 are structural proteins that self-assemble to form icosahedral capsids [9], while the fused product of ORFs E1 and E4 (E1^E4) is most abundant in the productive viral life cycle, coinciding with the onset of viral DNA amplification [10].

Among the HPV types, HPV16 (a member of species *Alphapapillomavirus* 9) is the most prevalent in cervical cancers. Intriguingly, and perhaps related to its prevalence, the HPV16 genome is polymorphic. Evolutionary analyses have revealed that the worldwide diversity of HPV16 genomes evolved for over 200,000 years [11], leading to five phylogenetic branches representing isolates from Africa, Europe, Asia and the Americas [12]. Furthermore, each branch can be further dissected into intratypic single nucleotide polymorphisms (SNPs) or variants differing in their host persistence and frequency of detection in human pre-cancers and cancers (reviewed in [13]). The tumourigenic differences of these SNPs have been ascribed largely to those within the E6 oncogene [14–17]. The Asian-American (AAE6) and European Prototype (EPE6) are common HPV16 genome variants which differ by six SNPs in their E6 genes, three of which are non-synonymous, leading to the 151-residue AAE6 protein differing by three amino-acids: Q14H, H78Y, and L83V [18] (with residue 14 and 83 being under Darwinian constraint [19]).

Epidemiological studies showed that the AAE6 genome variant is a higher risk factor for dysplasia as well as an earlier onset of invasive tumours than EPE6 [20–26]. As well, AAE6 has a greater transforming, migratory, and invasive potential than EPE6 when retrovirally transduced into primary human keratinocytes during recent long-term in vitro immortalization studies [27–30]. These results suggested that coding changes in E6 have strong mechanistic and functional consequences for infection and thus contribute to marked differences in cancer risk of HPV16 variants.

**Fig. 1:**
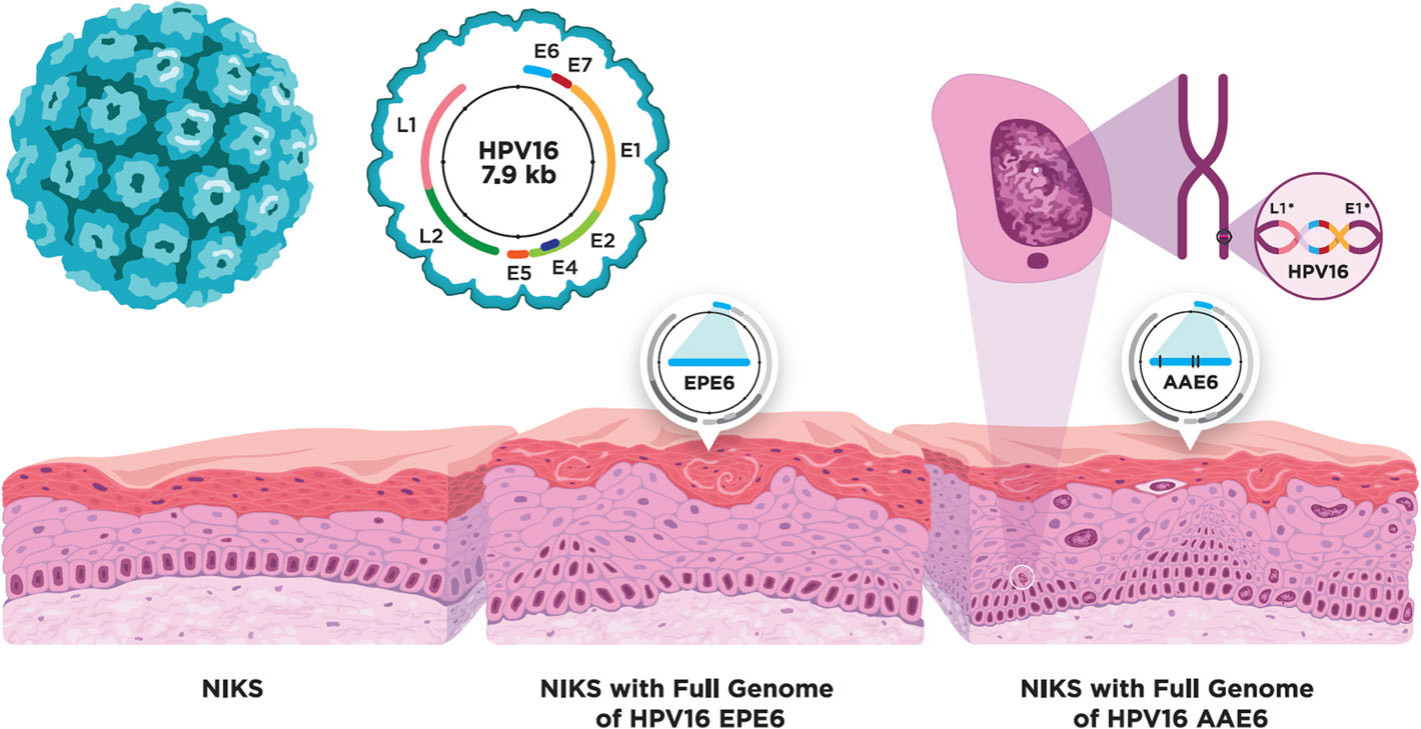
The HPV16 genome and our experimental epithelial model. Contained within the viral protein capsid (*top left*, not to scale relative to skin) is the 7.9 kb HPV16 genome, comprised of eight viral genes. Over a 14 day differentiation process we grew three-dimensional organotypic epithelia, or raft cultures, using near-diploid immortalized keratinocytes (NIKS) and primary human fibroblasts embedded in collagen-based dermal matrix. To permit the viral life cycle in this culture system we transfected the keratinocytes, prior to rafting, with complete viral genomes containing either the European Prototype or Asian-American variant of HPV16 E6 (EPE6 or AAE6, respectively). NIKS represented normal epithelia, NIKS with HPV16 EPE6 was a mild dysplasia (indicated by thickening and some suprabasal proliferation), whereas NIKS with HPV16 AAE6 was a moderate dysplasia (indicated by a greater number of suprabasal proliferating cells and abnormal cellular phenotypes, including micronuclei). Additionally, HPV16 viral integration was detected in AAE6 epithelia

To decipher the fundamental biology of HPVs and their tumourigenic features in a model system, the organotypic 3D infection model (raft culture) has the advantage of allowing reproducible and simultaneous epithelial differentiation and hence the occurrence of an active viral life cycle ([31]; Fig. 1). Thus, using engineered human epithelium resembling in vivo conditions based on near-diploid immortalized keratinocytes (NIKS [32]) we recently elucidated the phenotypic characteristics of both E6 gene variants in the context of the full HPV16 genome [31], building upon previous work on the effects of transduction with the E6 or E6/E7 genes only [27, 28, 33]. Using the organotypic model we observed that the AAE6 genome drives tumourigenesis by increasing epithelial proliferation, disrupting routine differentiation and apoptosis, evading the innate immune system and promoting immortalization [31]. Interestingly, we also observed that the differences in host epithelia histologically classified as mild keratinizing (EPE6) or moderate (AAE6) dysplasia were reflective of increased oncogene (E6 and E7) expression in AAE6 cultures and loss of productive life cycle (decreased E2, E1^E4, and L2). Together these observations lead us to suspect integration of the AAE6 viral DNA into the host genome [31], a common phenomenon during HPV-induced tumourigenesis (reviewed in [34]).

Here, to further advance our mechanistic understanding of the impact of these common but epidemiologically and clinically important E6 SNPs, we conducted an “-omics” analysis on the NIKS-based organotypic epithelia containing the HPV16 variants AAE6 and EPE6 (Fig. 1). Modern deep sequencing techniques have been used to study HPV [35–39], but only recently in the context of intratypic variants [40], and not using an organotypic epithelial model with full viral variant genomes. Instead, our complete approach allowed a comparison of these variants with regards to their integration capacity and subsequent transcriptional consequences in close to in vivo conditions, resulting in viral integration and a molecular signature of host chromosomal instability for AAE6 only.

## Results and discussion

### Viral integration in the HPV16 AAE6 but not EPE6 epithelium

To permit the viral life cycle in a raft culture system, we transfected the keratinocytes, prior to rafting, with complete viral genomes containing either the HPV16 EPE6 or AAE6 variant. A similar technique was used in a recent study to successfully study varicella zoster virus [41], providing a keratinocyte model and a “global” perspective of all changes in host transcription in response to a pathogen. As illustrated in Fig. 1, over a 14 day differentiation process, we observed that the NIKS were normal epithelia whereas NIKS with HPV16 EPE6 exhibited a mild dysplasia and NIKS with HPV16 AAE6 exhibited a moderate dysplasia.

**Fig. 2:**
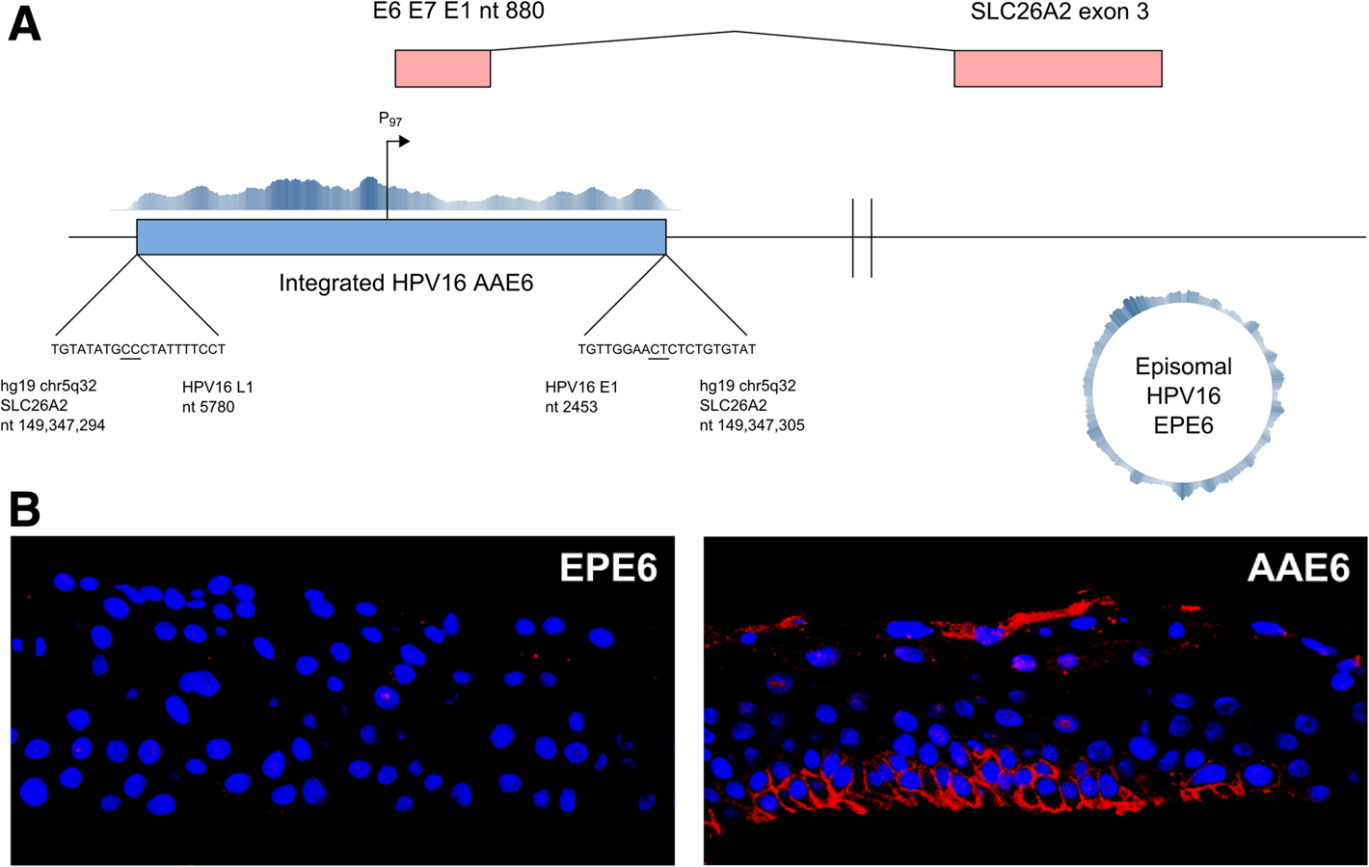
Characterization of viral integration and viral-human fusion transcripts in AAE6 epithelia. **a** Integration site schematic showing viral and human junctions, including nucleotide positions, the early promotor, as well as viral-human fusion transcript between HPV16 early region and SLC26A2 exon 3. A coverage plot above the integrated HPV16 genome demonstrates coverage across the junction sites within SLC26A2 (5482 reads across a 4978 nt assembly containing the AAE6 integrated form flanked by 200 nt of SLC26A2), while a circular coverage plot on the right shows the full episomal assembly of the EPE6 episomal form. **b** Immunofluorescence overlays of EPE6 and AAE6 raft cultures (400× magnification). Nuclei are indicated by blue DAPI staining while SLC26A2 is indicated by *red* fluorescence

To examine the HPV status of these cells we used the highly sensitive and high-throughput DNA capture and sequencing technique named Capt-HPV [42]. We prepared genomic DNA from epithelia of both EPE6 and AAE6. Then, after double capture on the HPV probes, we performed 2 × 151 nt paired-end sequencing (see Methods). As expected, we readily identified numerous HPV reads in both epithelial cultures. The sequencing reads of the E6 coding region confirmed the positive infection of the epithelia by the AAE6 and EPE6 variants. However, as we hypothesized [31], the physical genomic status of HPV was clearly different. In the EPE6 epithelia, the reads covered the entire HPV genome indicative of its episomal state (Fig. 2a) whereas only a fraction of the virus genome was detectable in the AAE6 epithelia, indicative of its integration into the host genome. Furthermore, in the case of EPE6, no human-viral junction reads were detected while the integrated AAE6 viral genome was truncated and several human-viral junction reads were identified in AAE6 epithelia. The integrated viral sequence was from nt 2453 (within HPV16 E1 gene) and nt 5780 (within HPV16 L1 gene) and thus includes the E6 and E7 oncogenes. Precisely, the insertion of the HPV16 AAE6 variant occurred between the nt position 149,347,294 and 149,347,305 of chromosome 5. Mechanistically, this is a simple “end-out” integration event with a typical two junction, co-linear (2J-COL) signature [42], associated with a very short 11 bp deletion of the host genome, and two overlapping nucleotides between viral and human sequence at each junction (Fig. 2a). Functionally, the insertion occurred within the 5q32 sub-band region, and more precisely, within the first intron of the SLC26A2 gene, approximately 13 kb upstream of its third exon.

Based on the Dr.VIS (Viral Integration Site) v2.0 database of HPV16 integration sites [43], this exact region (5q32) of integration is not frequent, but potentially recurrent as it was found in 2 out of 878 previously documented sites. The nearest fragile site was 13 Mb upstream of this integration site: FRA5C, 5q31.1. Since repeated regions might be prone to genome rearrangements and therefore prone to HPV integration, we scanned the adjacent regions using the UCSC hg19 genome browser RepeatMasker track for human repeat elements and found a nearby 158 bp long interspersed nuclear element (LINE): L1MB5 located from Chr5 nt position 149,347,143 to 149,347,300. Indeed, L1MB5-derived sequences have been documented as breakpoints, such as in the human genes HPRT [44], CYP2C [45], and in proximity of genes containing the ubiquitin ligase Mib-herc2 domain, which mediates Notch signalling [46]. Strikingly, this domain contains the Hect region, homologous to the E6-associated protein carboxyl terminus, raising the question of whether or not the underlying homology could play a role in this target site selection. Another, non-exclusive hypothesis is that the frequent hypo-methylation of LINE elements plays a role to facilitate access to the chromosomal DNA and associated genomic instability [47, 48]. Altogether, our three-dimensional organotypic cultures demonstrated that the HPV16 AAE6 variant had integrated into the host genome while the EPE6 variant remained episomal, suggesting an increased propensity towards integration due to AAE6. A previous study of HPV16 integration propensity with respect to the variants did not demonstrate a statistically significant difference (*P*-value = 0.28, two-tailed Fisher’s exact test) between EPE6 (3 episomal and 20 integrated cases) and the E-T350G variant (6 episomal and 16 integrated cases, responsible for one of the residue changes also found in AAE6: L83V) [49]. Only one tumour sample in their set contained the AA variant, therefore precluding a formal analysis of its propensity to integrate, but notably it was in integrated form.

### The HPV16 AAE6 epithelium has a unique transcriptional profile

**Fig. 3:**
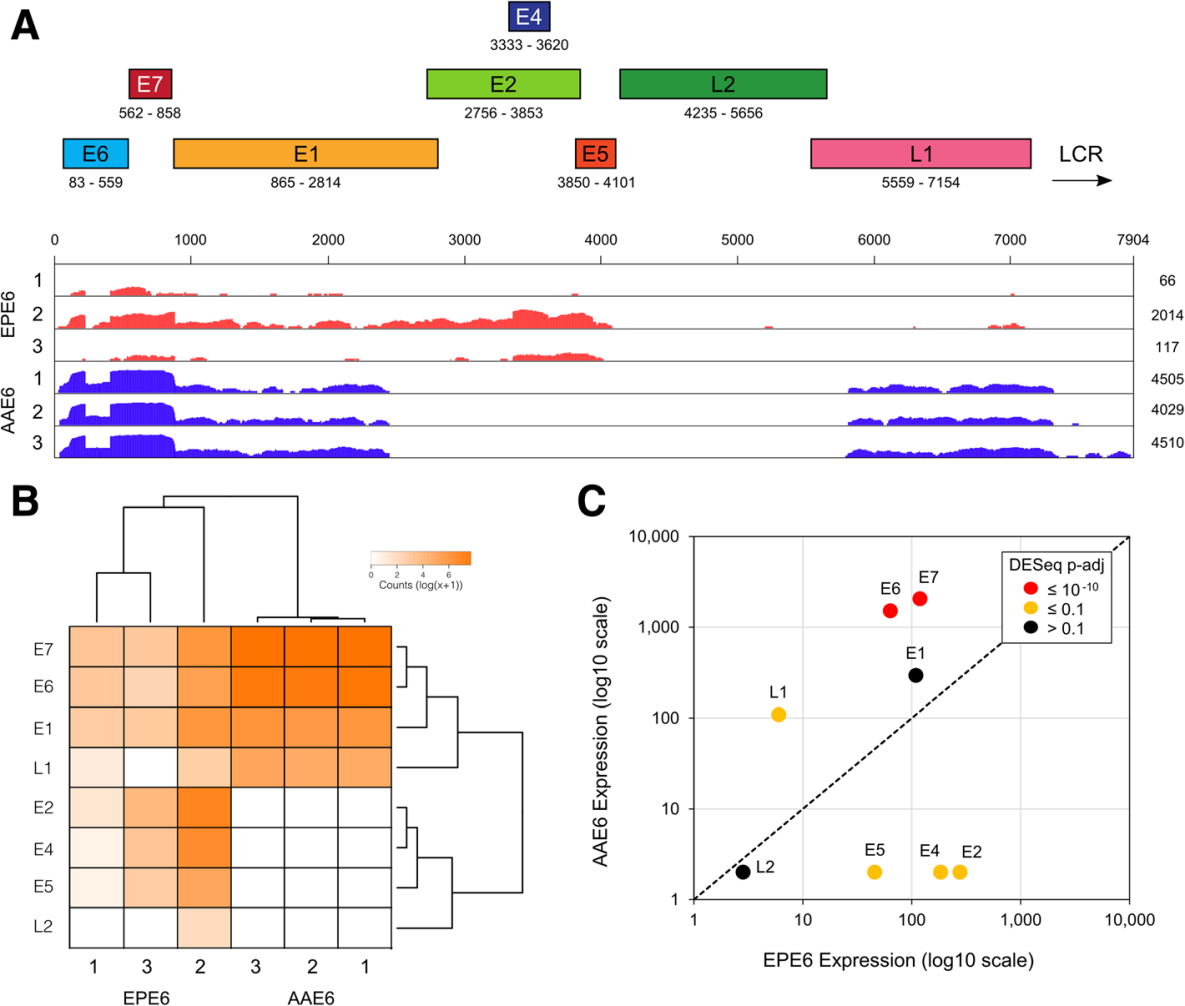
The HPV16 transcriptome in EPE6 and AAE6 organotypic rafts. **a** Linear viral gene map. Viral RefSeq ([GenBank: AF125673], HPV16 W12E genome) alignment from each individual raft culture was visualized using IGV [50]. The y-axis (coverage) is log_2_ scaled. Total number of viral reads are given on the right-hand side of each track. **b** Heatmap & clustering analysis of viral transcriptome on DESeq normalized counts: viral genes vs sample replicates. Two distinct sample clusters matched EPE6 and AAE6 replicates respectively, clustering independently of each other. Within the high-variability EPE6 cluster, replicate 1 and 3 were clustered together. Within the low-variability AAE6 cluster, replicate 1 and 2 were clustered together. As well, AAE6 epithelia converged on consistently high viral transcription (specifically E6/E7). From the viral gene perspective, two distinct clusters were identified: E6, E7, E1, and L1 in one, and E2, E4, E5, and L2 in another. Within the first primary cluster, E6 and E7 cluster close together, as expected given they are expressed together as a multi-cistronic transcript. E1 and L1 also cluster together, constituting the truncated transcripts on the periphery of the non-transcribed region within AAE6 samples. In the second primary cluster, E5 and L2 cluster together, independent of E2 and E4 which is transcribed only in EPE6 samples. E2 and E4 expression unsurprisingly clusters together given that E4 is contained within the E2 ORF. **c** Scatterplot of average viral gene expression for EPE6 samples (x-axis) and AAE6 samples (y-axis). The axes (DESeq normalized gene counts) are log_10_ scaled. Significant differential gene expression is denoted by marker colour. *Dashed line* represents equal expression

Another essential feature that may differentiate the behaviour of the HPV16 EPE6 and AAE6 variants is expression of the viral genome, viral-human fusion transcripts when integrated, as well as downstream host effects due to expression of the E6/E7 oncogenes. To assess these, we performed a genome-wide RNA-Seq analysis of the EPE6 and AAE6 epithelia using Illumina sequencing of total RNAs (see Methods), mapping first against our reference HPV16 W12E genome [GenBank AF125673]. Viral transcriptomes were visualized with the Integrative Genomics Viewer (IGV) [50], while viral gene counts and variant calls were performed using SAMtools [51]. The average sequencing depth of 40.4 million total reads per sample (~20 to 25 million fragments producing paired-end reads) was appropriate to detect the small proportion of total reads of both HPV variant genomes (~0.0001 to 0.01 %, Additional file 1: Table S1), while none were detected in the HPV-negative control epithelium. The variant-specific non-synonymous SNPs (relative to the reference HPV16 W12E genome) present in EPE6 (G350T) and AAE6 (G145T+C335T) were confirmed with depth of reads of 6× for EPE6 and with 14× to ~300× depth of reads for AAE6. Among the EPE6 epithelial samples, we detected few E6, E6*I (spliced transcript), E7, E1, E2, E1^E4, and E5 transcripts, with even fewer L2 and L1 reads, as confirmed by L2 RT-qPCR and L2 protein immunohistochemistry results from the same independent set of rafts reported previously [31]. Among the three individual epithelial raft cultures for EPE6 samples the viral transcriptional landscape appeared similar but the read coverage was higher in raft #2 due to an overall higher abundance of viral transcripts in this sample (Fig. 3a). In contrast, the transcriptional landscape for the three AAE6 samples was more homogenous (Fig. 3a), further emphasized in a clustered heatmap (Fig. 3b). Abundant full-length E6, E6*I, E7, and only truncated E1 and L1 transcripts were detected. Full-length E1, E2, E1^E4, and L2 reads were absent in AAE6 epithelia, consistent with the Capt-HPV data reported above and our previous RT-qPCR results and DNA copy number analyses on these molecules [31].

To quantitatively account for sample variance, we also performed differential expression analysis of the viral gene counts using DESeq [52]. DESeq software tests for differential expression in library size-corrected count data using a negative binomial distribution model. In agreement with our previous RT-qPCR results [31], we found significantly more E6 (24.05 fold higher, *P* < 10^−10^) and E7 (17.30 fold higher, *P* < 10^−10^) counts in triplicate AAE6 rafts in comparison to triplicate EPE6 rafts (Fig. 3c). Taken together, analyses of viral transcriptome data revealed that the AAE6 viral transcriptome significantly differs from that of EPE6 in a manner that is indicative of integration, with increased E6 and E7 levels [53–55]. Evidently, AAE6 transcriptome profiles are lacking E2 and have increased E6/E7 oncogene expression, perhaps due to loss of transcriptional repression by E2. We therefore reasoned that the increased levels of E6/E7 expression between the variants were ultimately due to their viral integration status, as we hypothesized in our phenotypic study, and confirmed by Capt-HPV, leading to a significant effect on the host transcriptome [31].

### Nature of viral-human fusion transcripts detected in HPV16 AAE6 epithelium

The integration of HPV16 genomes into host chromosomes is a frequent phenomenon associated with carcinogenesis, and not only modifies the expression of HPV-encoded E6 and E7 oncogenes (Fig. 3a), but can also trigger the expression of fusion viral-human mRNAs [34, 56]. Since the virus can integrate into a variety of positions in the human genome, these fusion transcripts are specific to each integration site. In recent years, following the introduction of high-throughput sequencing techniques, multiple softwares for detecting pathogen sequences in host sequence data have become available [38, 57–63]. Here, to identify the viral-human fusion transcripts expressed in our epithelia, we used the ViralFusionSeq (VFS) software [61, 64]. VFS was chosen over alternatives due to its optimization for RNA-Seq data from the Illumina platform, the ability to define our own reference virus genome, as well as the full suite of fusion transcript discovery techniques it uses. Using this technique, only the AAE6 rafts yielded viral-human fusion transcripts (Table 1), providing further evidence of viral integration as well as its transcriptional impact.

**Table 1:** Integration loci detected by ViralFusionSeq

Sample	Mapped human transcript†	Gene description	Chromosome location	HPV transcript breakpoint(s)‡
AAE6	SLC26A2	Solute carrier family 26, member 2	5q32	E1, L1
	PDE6A	Cyclic GMP- Phosphodiesterase 6A alpha subunit	5q32	E1, L1
EPE6	None	–	–	–
NIKS	None	–	–	–

Viral-human fusion transcripts were discovered using ViralFusionSeq’s [61]: clipped-sequence (CS) and read-pair (RP) modules. Detected by at least 1 RP and CS event (†). As detected by CS method (‡). VFS uses two methods to detect viral-human fusion transcripts. The Clipped-Seq (CS) method detects viral fusion transcript breakpoints with a read that maps to both viral and human sequences, while the Read-Pair (RP) analysis detects transcripts with read ends mapped separately to the viral and human genome [61]. We required candidate viral fusion transcripts to be supported by at least 1 CS and 1 RP event in order to improve its stringency [64]. Although RP events were more abundant in our samples, CS analysis provided single-base resolution of viral-human fusion transcript breakpoints. In particular, we identified an average of 1.33 +/− 1.53 CS transcripts in EPE6 and 7.66 +/− 6.66 in AAE6. We detected no RP transcripts in EPE6, while 118.66 +/− 7.23 were found in AAE6 rafts. While one RP transcript was detected in a NIKS control culture, this read was not confirmed by the CS method of VFS and therefore not considered as a valid event

In accordance with the structure of the HPV integration, the transcript breakpoints mapped to either the E1 or L1 HPV16 ORF. Alternative splicing was detected with the viral nucleotide position at the fusion site of one class of the viral-human fusion transcripts (Fig. 3a): nt 880 (splice donor, SD) in the E1 gene [65]. This is the same SD site for the E1^E4 splice transcript typically expressed in the late stage of the viral life cycle [66], and previously shown to be expressed in our EPE6 epithelia [31]. HPV16 viral-human fusion transcripts are often detected with a breakpoint at this natural splice donor site [56, 67, 68], and the coverage plot for AAE6 shows decreased coverage for transcripts downstream of this E1 SD site, supporting the hypothesis of alternative splicing. With respect to the L1 breakpoints, the typical L1 splice acceptor (SA) site is at nt 5639 [65], but notably in our study, the viral-human fusion transcripts here had a putative downstream SA site at nt 5778. Interestingly, the coverage plot of the viral transcriptome shows nt 5778 as the site where L1 coverage begins to be detected in AAE6 rafts (Fig. 3a), so we reasoned that this discrepancy in SA site could be due to either a cryptic SA site in the HPV16 W12E genome (although not found previously in the literature) or simply due to integration truncating the upstream region of L1.

Next, we mapped the human portion of the fusion transcripts using VFS’s clipped-seq (CS) and read-pair (RP) methods. Confirmed by both these methods, two fusions mapped to the human chromosome location 5q32, occurring within the solute carrier family 26 (anion exchanger), member 2 (SLC26A2) and phosphodiesterase 6A, cGMP-specific, rod, alpha (PDE6A) human ORFs (Table 1). Strikingly, along with detection of fusion transcripts with these genes, we detected a significant increase in the expression of human genes from this region in AAE6 epithelia compared to normal epithelia, namely SLC26A2 (114.19 fold increase, *P* = 2.14 × 10^−173^) and colony-stimulating factor 1 receptor (CSF1R, 407.82 fold increase, *P* = 4.70 × 10^−112^, which was only detected as RP fusion reads by VFS, and not confirmed by CS). This observation is in agreement with others who have found that, in numerous cervical carcinomas across multiple high-risk HPV types, HPV integration leads to an increase in the expression of genes adjacent to integration loci [69]. To explain the molecular basis of this cis-effect, it has been proposed to be the result of viral promotor-driven expression or somatic genome amplification at the integration site [70, 71]. In the present case, this last hypothesis is unlikely because the AAE6 integration produced a clean 11 bp deletion of the target region that led to two co-linear viral-human junctions (2J-COL), which is not associated with gene amplification [42].

Functional human fusion proteins can be formed due to chromosomal translocations in cancer cells [72]. The elucidation of novel protein-coding viral-human fusion transcripts is particularly intriguing due to their potentially functional roles within host cells. Using immunofluorescence for the expressed portion of the SLC26A2 protein in formalin-fixed and paraffin embedded (FFPE) rafts, we determined that SLC26A2 protein expression was aberrantly high in AAE6 compared to EPE6, supposedly as a result of its viral-human fusion and increased transcription (Fig. 2b). This translated fusion protein contains exon 3 of the transmembrane protein SLC26A2, previously known as diastrophic dysplasia sulfate transporter (DTDST) [73], which encodes the carboxy-terminal cytoplasmic sulfate transporter and anti-sigma factor (STAS) domain [74]. We cannot find any evidence in the literature of this unique viral-human fusion protein in other HPV-integrated samples. Overall, these chimeric molecules are unique for each sample and to the specific integration site, with presently unknown effect on host cell functions, an aspect to be further researched due to its importance for understanding mechanisms of tumourigenesis as well as in the emerging field of personalized medicine.

### The HPV16 AAE6 epithelium reveals a signature of chromosomal instability conducive to host genome integration

Integration of HPV DNA into the host genome is considered to be a key factor for cervical cancer development [67, 75, 76], but the cellular events that initiate the integration process (and selection of insertion sites) remain to be better understood. A reasonable hypothesis is that the integration is triggered by a rare and stochastic target site event, such as a replicative fork stalling or an accidental chromosome double-strand break, leading to an ultimate use of the viral DNA for repair via recombination, template switching (FoSTeS) and/or microhomology-mediated break-induced replication (MMBIR) ([42, 71, 77], and references within each). Indeed, infections with pathogens can cause chromosomal instability by inactivating the host DNA damage response [78]. For HPV, this has been linked to the expression of both HPV16 E6 and E7 oncoproteins, affecting the infected cell’s genome integrity [79–82]. A model of early carcinogenesis due to HPV16 E6 and E7 suggests that this chromosomal instability is caused by uncontrolled proliferation, leading to an insufficient nucleotide pool that cannot support normal replication [83]. Alternatively, E6 alone, through the inactivation of p53, can promote chromosomal instability, at least during early onset of carcinogenesis [84]. Presently, HPV16 AAE6 demonstrated enhanced integration propensity over EPE6 and exhibited increased E6 and E7 oncogene expression, which is in accordance with elevated E6 and E7 levels reported in other studies [53–55]. This enhanced integration ability is based on AAE6’s greater proliferation ability, leading to chromosomal instability. The underlying mechanism of its increased cell growth is the result of a deregulated sugar metabolism (Warburg effect), as we reported previously [28] and currently under study (Cuninghame et al., in preparation: unpublished observations).

**Fig. 4:**
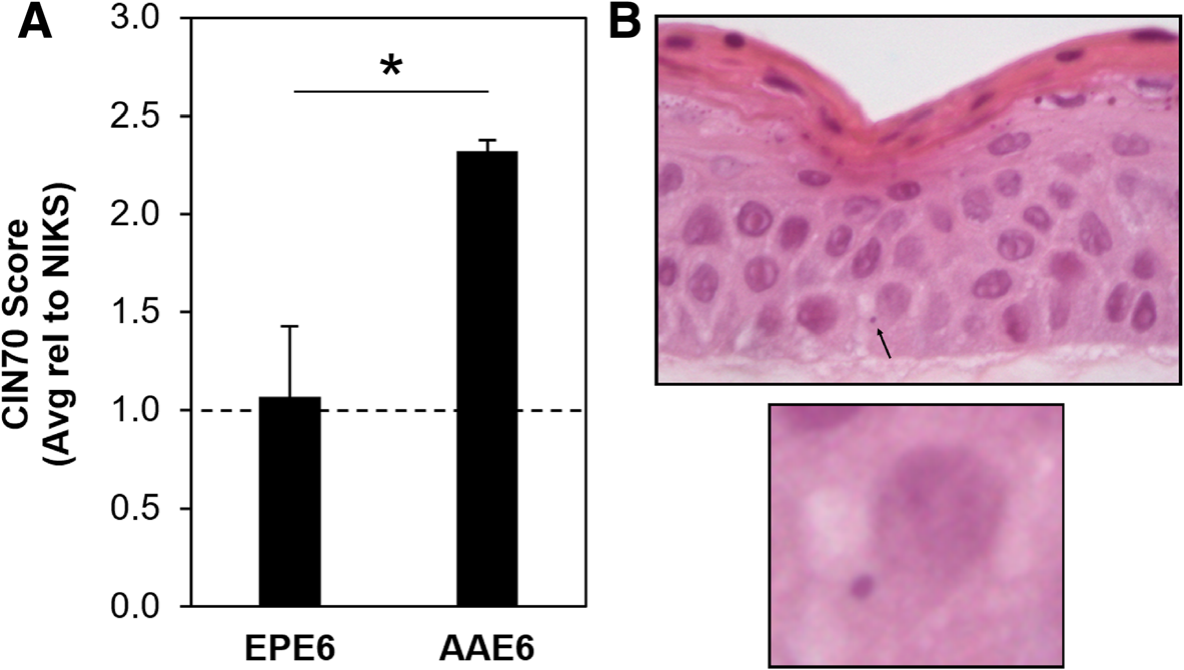
Chromosomal instability signature and micronuclei in AAE6 epithelia. **a** The CIN70 score relative to HPV-negative NIKS was significantly higher in AAE6 compared to EPE6 epithelia (2.32 fold higher, *P* = 0.02 by Welch’s *T*-test). Mean values are shown with error bars representing standard deviation (*n* = 3). Statistical significance (*P* < 0.05) denoted by “*”. **b** Haematoxylin and eosin micrographs of FFPE AAE6 epithelia, 400× cropped, micronuclei indicated by *arrow*. Close-up shows micronucleus and normal-sized nucleus within same cell

To assess the host chromosomal instability in our HPV16 variant epithelia, we examined our RNA-Seq data to detect the CIN70 gene expression signature [85], which has been applied as a prognostic marker in cervical cancer [86] and more generally as a significant indicator to predict clinical outcome across multiple cancer types [85]. This signature is derived from 18 gene expression datasets (with genes ranked based on their correlation to functional aneuploidy). The CIN70 score relative to HPV-negative NIKS was significantly higher in AAE6 compared to EPE6 epithelia (2.32 fold higher, *P* = 0.02 by Welch’s *T*-test), indicating a signature of host chromosomal instability in AAE6 epithelia (Fig. 4a). Furthermore, as a morphological sign of chromosomal instability, we detected micronuclei (MN) in AAE6 but not EPE6 or NIKS FFPE H&E-stained epithelia (Fig. 4b). MN were reported to be present in higher grade cervical intraepithelial neoplastic lesions and invasive cervical cancer [87] and mechanistically have been associated with hallmarks of genomic instability [88].

### HPV16 AAE6 epithelium exhibits a proliferating phenotype as a consequence of viral integration into the host genome

**Fig. 5:**
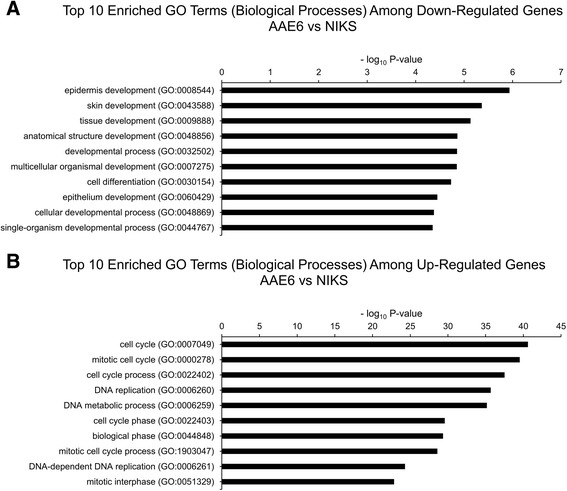
Gene Ontology (GO) terms enriched in highly significant differentially expressed genes in AAE6 vs. NIKS. The Term Enrichment Service available on the AmiGO 2 website [104] was used to determine enriched GO (biological process) terms among (**a**) down-regulated and (**b**) up-regulated genes. Only the top ten GO terms are shown for each. See Additional file 4 for discussion

**Fig. 6:**
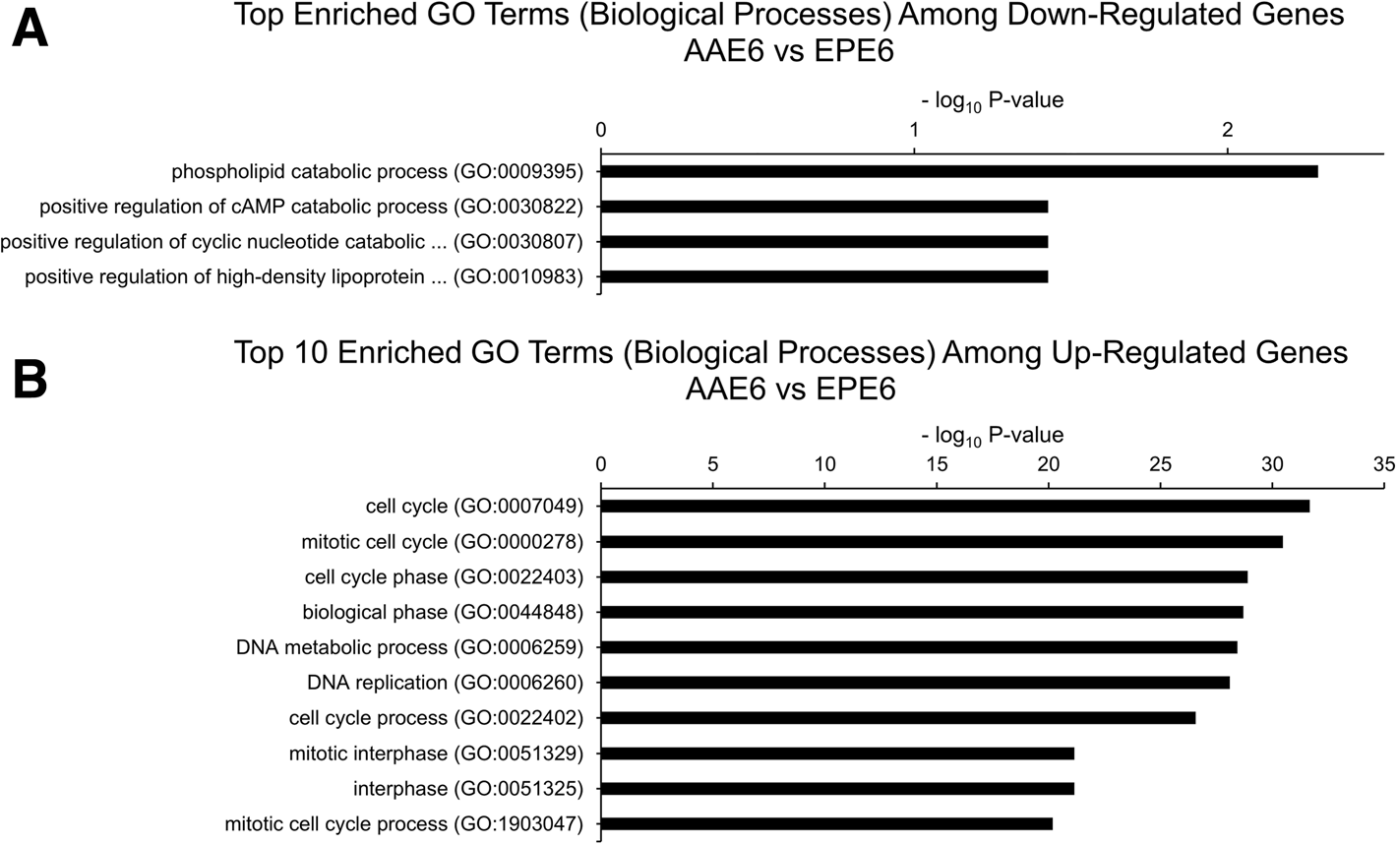
Gene Ontology (GO) terms enriched in highly significant differentially expressed genes in AAE6 vs. EPE6. The Term Enrichment Service available on the AmiGO 2 website [104] was used to determine enriched GO (biological process) terms among (**a**) down-regulated and (**b**) up-regulated genes. Only the top ten GO terms are shown for each. See Additional file 4 for discussion

**Fig. 7:**
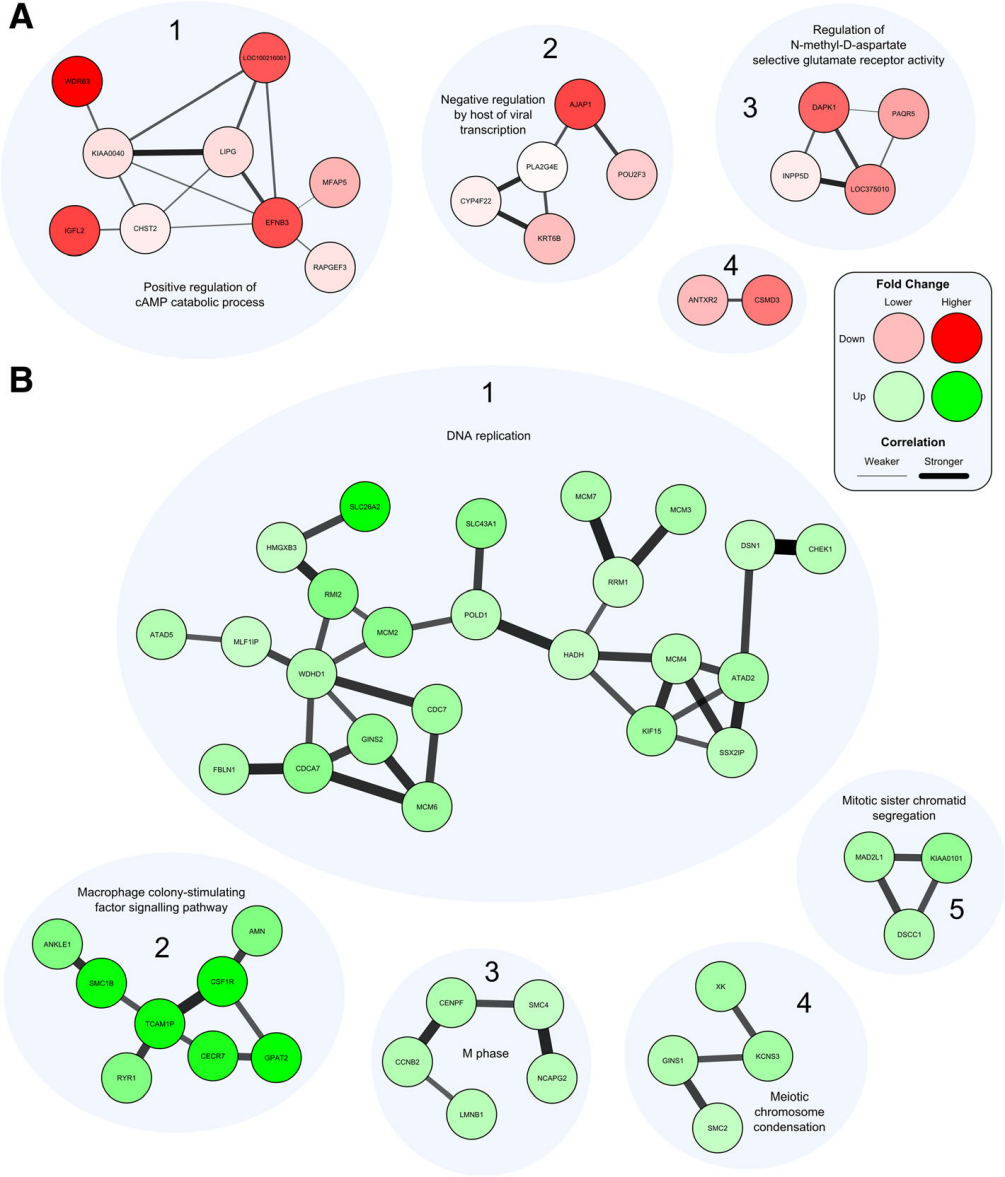
Co-expression networks of highly significant (**a**) down-regulated and (**b**) up-regulated genes in AAE6 vs. EPE6. **a** Four discrete clusters of down-regulated and co-expressed genes were observed. Only co-expressed genes with a Pearson correlation coefficient greater than 0.95 are shown. Clusters are labelled by number and functionally annotated with their significantly enriched biological process. Nodes = gene, denoted by gene symbol; node colour = white to red with down-regulation (fold change) in AAE6 from EPE6; edge thickness = increases with Pearson correlation coefficient. **b** Five discrete clusters of up-regulated and co-expressed genes were observed. Only clusters co-expressed genes with a Pearson correlation coefficient greater than 0.996 and are shown, to narrow down the number of genes displayed. Clusters are labelled by number and functionally annotated with their significantly enriched biological process. Nodes = gene, denoted by gene symbol; node colour = white to green with up-regulation (fold change) in AAE6 over EPE6; edge thickness = increases with Pearson correlation coefficient. See Additional file 4 for discussion

**Fig. 8:**
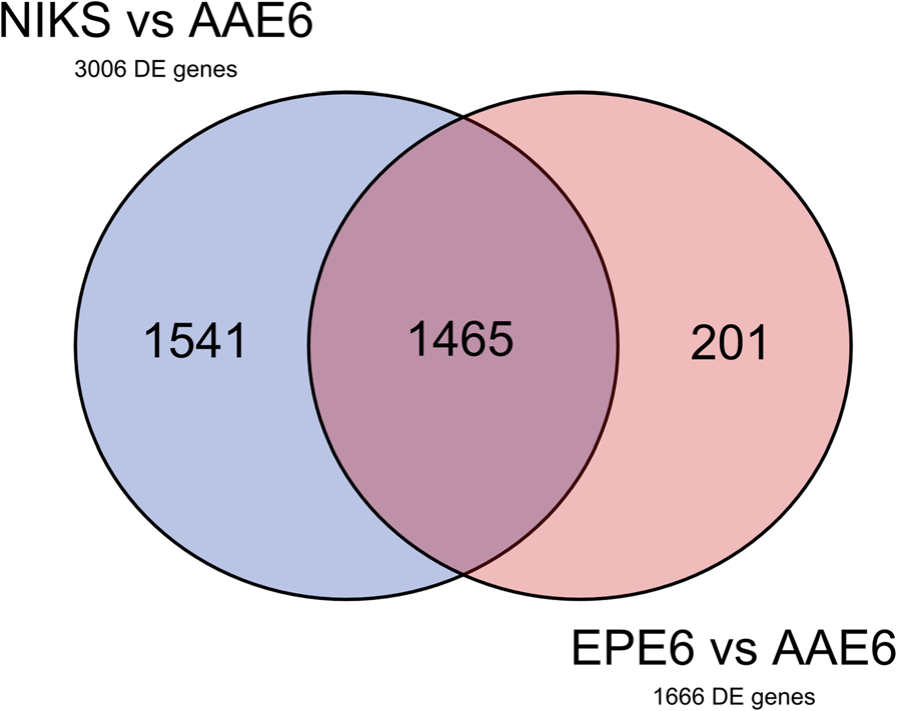
Venn diagram of differentially expressed genes common and unique to each pairwise comparison. Of the 3006 differentially expressed (DE) genes in NIKS vs AAE6 and the 1666 differentially expressed (DE) genes in EPE6 vs AAE6 there were 1541 genes unique to the NIKS comparison, 1465 common between them, and 201 unique to the EPE6 comparison. No genes were up-regulated in one set of a pair-wise comparison (either NIKS vs EPE6 or EPE6 vs AAE6) while down-regulated in the other

More broadly, our RNA-Seq data led us to examine global changes in host gene expression. Our previous study demonstrated enhanced tumourigenesis by the full HPV16 genome with AAE6 [31], while another study presented altered gene expression by the AA variant [89]. Work by other groups have studied the downstream pathways in the AA variant [90, 91], and have utilized high-throughput techniques to investigate genetic variation within HPV16 [39, 40, 92], but this is the first study investigating the downstream pathways affected by the HPV16 variants in an organotypic epithelial model using next-generation sequencing. We hypothesized two scenarios that can be associated with these findings and analyzed in our present study: i) the global gene expression profile within AAE6-infected epithelium would differ significantly from that of EPE6 and ii) significant gene expression differences in the host due not only to the actions of the viral oncogenes E6 and E7, but also as a result of integration [56]. A global “-omics” technique, RNA-Seq, was required to sufficiently address our hypotheses around the functional relevance of the AA variant in epithelia. We assessed host differential gene expression using DESeq [52] to determine how it reflected the unique viral gene expression profiles induced in human epithelium undergoing differentiation. Strikingly, NIKS, which contain no virus genome, had zero significant differentially expressed genes compared to EPE6, at a false-discovery rate (FDR) of 10 % (Additional file 2: Figure S1). NIKS to AAE6 had 3006 significant differentially expressed genes (Additional file 2: Figure S2, Additional file 3 for list of differentially expressed genes between NIKS and AAE6). Of these genes, 1312 were down-regulated while 1694 were up-regulated in AAE6 compared to NIKS. The lack of any differentially expressed genes between NIKS and EPE6 organotypic epithelial cultures was surprising, but consistent with the similarity between the NIKS and EPE6 cultures monitored with respect to basal and suprabasal keratinocyte proliferation assessed by BrdU-incorporation, p53 and p16^INK4A^ by immunohistochemistry and IFN-κ by RT-qPCR [31]. Phenotypically, these results suggest that the episomal expression of the EPE6 variant in our model does not have a significant tumourigenic effect. Since our 3D culture model specifically captures early tumourigenesis, with only a 2-week growth period and low initial viral copy number, very small gene expression differences in a homogenized epidermal sample are not expected to be easily detected with global transcriptomic techniques. On the other hand, AAE6 significantly perturbed a high number of human genes, demonstrating its ability to cause a wide-range of host molecular changes consistent with tumourigenesis. Compared to EPE6, AAE6 had 1666 significant differentially expressed genes (Additional file 2: Figure S3, Additional file 3 for list of differentially expressed genes between EPE6 and AAE6). Of these genes, 666 were down-regulated while 1000 were up-regulated in AAE6 compared to EPE6. Additional discussion of the top-ten most significant down- and up-regulated genes for each pair-wise comparison is provided in Additional file 4. To further investigate the differential gene expression data we applied two additional bioinformatics analyses: gene ontology (GO) biological process term enrichment (Additional file 5 for GO output, Figs. 5 and 6), as well as co-expression analysis and visualization using networks (Fig. 7). Finally, we also compared the pair-wise lists of differentially expressed genes to determine the number of common and unique genes among each set (Fig. 8): 1541 genes unique to the NIKS comparison, 201 unique to the EPE6 comparison, and 1465 common between them. Overall, these bioinformatics analyses highlight the global effects of AAE6 on host epithelia due to its integration event, increased E6/E7 expression, and perhaps in part functional differences due to the AAE6 oncoprotein itself: increased proliferation and decreased differentiation.

## Conclusions

We have systematically characterized the viral integration process of a common high-risk HPV16 variant and its consequences for the affected host cell. This and earlier work lend themselves to propose a model of increased tumourigenicity in human keratinocyte epithelia where AAE6’s enhanced ability to proliferate leads to chromosomal instability. In such an environment, the host genome may be susceptible to viral integration subsequently increasing E6/E7 oncogene expression and ultimately driving additional tumourigenic changes. Previously, we performed phenotypic studies of the EPE6 and AAE6 variants in a 3D raft model of early carcinogenesis [31] and determined the functional differences of these variants in longitudinal monolayer cell cultures [27–30]. While necessary for studying the viral life cycle, limitations of the current organotypic model are the lack of immune components, vasculature, and the complexity of tissue heterogeneity that arises. Our current study builds on the foundation of these investigations. We have applied a wide range of molecular analyses, creating a framework which can benefit future virus-host interaction studies with various organotypic cell culture models. A variant-specific integration is worth reporting and should be further investigated, with additional samples from independent donors, as it represents a new paradigm in HPV variant biology. Here we report a viable integration mechanism in a robust viral life cycle model for AAE6. The findings of the current and other studies reported by us [27–31], and others [89–91], are consistent with cancer epidemiology studies demonstrating that the HPV16 AA variant is a higher risk factor for high-grade intraepithelial neoplasia and progression to invasive cervical cancer [22–24, 89]. In the future, HPV variant genotyping could be used as a clinical prognostic factor for patient-centered health services, while the role of individual host genomics on integration, including characterization of integration sites, will be important to consider for personalized medicine approaches.

## Methods

### Cell lines

As described by us previously [31], we used the Normal/Near-Diploid Immortalized Keratinocytes (NIKS) cell line [32] to establish 3D organotypic epithelia cultures. These spontaneously immortalized cells were originally derived from neonatal human foreskin and are non-tumourigenic, though contain an additional long arm piece of chromosome 8 (8q). In monolayer they are grown on mitomycin-C-treated Swiss mouse J2/3T3 fibroblast feeder layers [32], while primary human foreskin fibroblasts (ATCC CRL-2097) are incorporated into the dermal equivalent of organotypic NIKS cultures [31].

### Detection of integrated papillomavirus sequences by next-generation DNA-Seq: Capt-HPV

DNA-Seq was used to confirm the presence and location of the viral integration sites in the human genome using DNA extracted from formalin-fixed paraffin embedded (FFPE) samples which had been prepared previously [31]. DNA was extracted using the DNeasy Blood and Tissue Kit (QIAGEN, Cat# 69504) with the recommended pre-treatment for FFPE samples and the optional RNase treatment. To overcome the limitations of traditional techniques, such as DIPS-PCR (Detection of Integrated Papillomavirus Sequences by ligation mediated PCR), we used an unbiased and state-of-the-art next-generation DNA sequencing technique for detecting HPV viral integration sequences in our samples [42]. Library preparation, sequence capture, and high-throughput sequencing was carried out at the Institut Curie on an Illumina MiSeq platform with a V2 Nano chip (~1 × 10^6^ total reads) with 2 × 151 base pair read length. Analysis of sequencing data was performed using the Galaxy platform [93–95], with the primary goal of detecting the viral-human junction site locations. Packages used were FASTQ Groomer [96], Bowtie2 [97], Picard MarkDuplicates [98], SAMtools BAM-to-SAM and Filter SAM [51].

### RNA-Seq library preparation and sequencing

Isolation of high-quality total RNA from the epithelium of organotypic keratinocyte cultures containing full-length HPV16 E6 variant genomes, European Prototype (EPE6) and Asian-American (AAE6), was described previously [31]. Our keratinocyte model was grown for 14 days to allow simultaneous epithelial differentiation and occurrence of an active viral life cycle. Total RNA for EPE6, AAE6, and HPV16 negative cultures (NIKS), three organotypic raft cultures (*n* = 3) each, were sent for library preparation and sequencing at The Centre for Applied Genomics, Hospital for Sick Children, Toronto, Canada. RNA-Seq libraries were prepared by Illumina TruSeq® RNA Sample Preparation kit followed by sequencing using an Illumina HiSeq® 2500 platform with Illumina v3 chemistry. One lane of multiplexed, paired-end, 2 × 101 base pair sequencing was performed with nine samples: yielding an average of 40.4 million total reads (~20 to 25 million fragments) per sample (Additional file 1: Table S2).

### Viral variant read alignment, mapping, and coverage plotting

The human papillomavirus type 16 W12E isolate genome [GenBank: AF125673] [54, 99] was used as a viral reference sequence since it was the parental sequence modified by site-directed mutagenesis to generate the EPE6 and AAE6 viral genomes used in this study [31]. Only the three non-synonymous nucleotide changes differentiated EPE6 and AAE6 genomes: EPE6 was made by mutating the parental W12E genome at G350T while AAE6 was mutated at G145T and C335T. Prior to alignment and mapping, Bowtie2 [97] was used to build a reference index for HPV16 using the AF125673 W12E isolate RefSeq. TopHat2 [100] was used for alignment to our viral RefSeq. Variant-specific non-synonymous SNPs were confirmed by variant calling with SAMtools [51]. The Broad Institute’s Integrative Genomics Viewer (IGV) [50] was used to visualize alignment coverage for each sample. Gene-level counts of the HPV16 W12E ORF’s were generated using SAMtools [51], and normalized with library-size correction factors using the Bioconductor project DESeq [52] in the statistical environment R [101]. DESeq was also used for differential viral gene expression analysis. DESeq uses a default false discovery rate (FDR) of 10 % for its binomial statistical inference tests to determine differentially expressed genes. Clustered heatmaps of normalized viral gene counts were generated using the gplots package [102].

### Identification of viral-human fusion transcripts

ViralFusionSeq (VFS) [61] was used, with default parameters, to identify any viral-human fusion transcripts in each of our sample RNA-Seq datasets. As with viral alignment by TopHat2 (described above), the W12E genome was used as a reference sequence for VFS. Briefly, VFS is a Perl script that searches in high-throughput sequencing data (RNA or DNA-Seq) for viral-human fusion transcripts, which are present as a result of viral integration events into host DNA. This software uses read pair (RP) and clipped sequences (CS) to accurately discover and identify viral-fusion sequences [61]. Additionally, VFS is able to reconstruct fusion transcripts by a targeted *de novo* assembly process. These methods allow us to identify, with single-base resolution, viral-human fusion transcripts present within our epithelial cultures. Viral-human fusion transcripts were compared to known HPV16 integration sites and fusion transcripts with assistance from the database of disease related viral integration sites (Dr. VIS v2.0, [43]).

We sought to perform protein-level confirmation of highly expressed viral-human fusion transcripts containing exons from human targets SLC26A2 and CSF1R. SLC26A2 protein expression was detected in raft cultures by immunofluorescence, as described previously [31]. Based on the viral-human fusion RNA-Seq data, the primary antibody (rabbit polyclonal, 1:500 dilution, Bethyl Laboratories Inc., Cat. No. A304-467A) was chosen to have specificity for translated exon 3 (epitope between amino acid residue 689 and 739). Although also highly up-regulated, no suitable commercial antibody was found for CSF1R exons 20 to 22.

### Human read alignment, mapping, and count generation

Read alignment, mapping, and count generation for the human reference genome (hg19, UCSC nomenclature for GRCh37) was performed by The Centre for Applied Genomics, Hospital for Sick Children, Toronto, Canada. TopHat2 [100] was used for RefSeq while gene- and exon-level counts were generated using HTSeq [103]. Number of reads and percentage of human RefSeq reads defined as aligned, exon, and exon-exon are reported in Additional file 1: Table S2 for each sample analyzed.

### Differential expression analysis of human transcriptome

Differential analysis of pair-wise human gene-level counts between NIKS and EPE6, NIKS and AAE6, and EPE6 and AAE6 were performed using the Bioconductor project DESeq [52] package implemented in the statistical environment R [101]. Raw gene counts from HTSeq were first normalized by estimating the sample library sizes (Additional file 1: Table S3) and applying the size-factor correction to all counts within a given sample. A dispersion plot was made to visualize the variance estimation step prior to differential expression inference (Additional file 2: Figure S4). A clustered heatmap with hierarchical dendrograms was used to show overall sample and biological replicate clustering: the gene expression profile of AAE6 samples was distinct from EPE6 and NIKS (control) samples (Additional file 2: Figure S5). Although EPE6 replicate 3 and NIKS replicate 1 cluster outside of their specific sample group, viral RNA-Seq analysis has confirmed these sample ID’s are correct, and that their grouping is likely a result of the minor host transcriptomic difference between NIKS and EPE6 cultures. DESeq uses a default false discovery rate (FDR) of 10 % for its binomial statistical inference tests to determine differentially expressed genes. However, for downstream analyses of down- and up-regulated genes we used a more stringent adjusted *P*-value cut-off of 10^−5^.

### CIN70 scoring and micronuclei detection

Host chromosomal instability was assessed, using normalized human gene count data from our RNA-Seq experiments, by calculating a CIN70 gene expression signature score [85] for EPE6 and AAE6 relative to NIKS epithelia. For each of the 70 genes, a normalized human gene count ratio was calculated for all EPE6 and AAE6 samples relative to the average of the NIKS samples. Relative ratio values were then averaged for all 70 genes in each sample and a Welch’s *T*-test, for unequal variance, was used to determine whether there was a statistically significant difference in host chromosomal instability signature between EPE6 and AAE6 epithelia. We used a significance level of *P* < 0.05. As a morphological assessment of chromosomal instability we screened haematoxylin and eosin-stained sections from formalin-fixed and paraffin-embedded NIKS, EPE6, and AAE6 epithelia for micronuclei (MN). These aberrant nuclei structures [88] were detected using light microscopy with high-magnification (at least 400×).

### Gene set enrichment analysis and networks

Enrichment of host biological processes of differentially expressed human genes was determined using the Gene Ontology (GO) Term Enrichment Service hosted on the AmiGO 2 website [104]. Only biological processes were included. Terms were considered significantly enriched if the Bonferroni-corrected *P*-value was less than 0.05. To aid in the visual interpretation of down- and up-regulated gene sets, co-expression networks were constructed with Cytoscape software [105]. Pearson correlation coefficients were calculated for each gene-gene pairwise comparison in highly significant down- and up-regulated genes between AAE6 and EPE6 (Additional file 6 for down- and up-regulated gene-gene pairwise comparisons, respectively). Pearson correlation coefficient cut-offs used for networking were selected strategically to produce small distinct clusters of genes, since setting the threshold too low results in all nodes connected, and setting the threshold too high results in a lack of clusters.

## Additional files

Additional file 1: Viral and human read tables. **Table S1.** Viral reads summary. Overall, viral reads make up ~0.0001 to 0.01 % of the total reads, while human reads make up 80 to 85 % of the total reads (the remaining reads are unmapped, to either viral or human sequences). **Table S2.** Human RefSeq alignment statistics for all samples. NIKS were HPV16 negative organotypic keratinocyte cultures while EPE6 and AAE6 were cultures containing the full genome of HPV16 with either European Prototype E6 or Asian-American E6 variants, respectively. “Aligned” refers to reads overlapping exons, “Exon” refers to reads completely within an exon, and “Exon-Exon” refers to reads overlapping exon junctions. **Table S3.** Human library size factor for all samples. Library size factors derived from DESeq [52]. (DOCX 15 kb)
Additional file 2: DESeq plots. **Figure S1.** Plot of normalized mean counts versus log_2_ fold change for the contrast NIKS versus EPE6. Red points represent genes that have significant differential expression between the two conditions (false-discovery rate of 10 %, adjusted *P* < 0.1). No genes were significantly differentially expression between NIKS and EPE6. **Figure S2.** Plot of normalized mean counts versus log_2_ fold change for the contrast NIKS versus AAE6. Red points represent genes that have significant differential expression between the two conditions (false-discovery rate of 10 %, adjusted *P* < 0.1). In total, 3006 genes were significantly differentially expression between NIKS and EPE6. **Figure S3.** Plot of normalized mean counts versus log_2_ fold change for the contrast EPE6 versus AAE6. Red points represent genes that have significant differential expression between the two conditions (false-discovery rate of 10 %, adjusted *P* < 0.1). In total, 1666 genes were significantly differentially expressed between NIKS and EPE6. **Figure S4.** Empirical and fitted dispersion values plotted against the mean of the normalized human gene-level counts. Red line represents fitted dispersion over the empirical values (black dots). **Figure S5.** Heatmap of Euclidean distances between human gene-level counts of samples. Heatmap and clustering was performed after DESeq variance-stabilizing transformation of human gene-level count data. (DOCX 204 kb)
Additional file 3: DESeq output. Significant differential expression output for NIKS and AAE6 contrast as well as EPE6 and AAE6 contrast. (XLSX 430 kb)
Additional file 4: Follow-up discussion of host expression analysis. Additional discussion of differential gene expression analysis, pathway-level enrichment, and co-expression networks. **Tables S4**-**S7.** top-ten most significant down- or up-regulated genes in AAE6 compared to NIKS or EPE6. (DOCX 30 kb)
Additional file 5: GO output. Significantly enriched GO terms (biological processes) for NIKS and AAE6 contrast as well as EPE6 and AAE6 contrast. (XLSX 28 kb)
Additional file 6: Pearson correlations Pearson correlation coefficients for gene-gene pairwise comparisons of down- and up-regulated genes for EPE6 and AAE6 contrast. (XLSX 298 kb)
