# Erratum to: Functional variants of human papillomavirus type 16 demonstrate host genome integration and transcriptional alterations corresponding to their unique cancer epidemiology

**DOI:** 10.1186/s12864-016-3379-6

**Published:** 2016-12-12

**Authors:** Robert Jackson, Bruce A. Rosa, Sonia Lameiras, Sean Cuninghame, Josee Bernard, Wely B. Floriano, Paul F. Lambert, Alain Nicolas, Ingeborg Zehbe

**Affiliations:** 1 Probe Development and Biomarker Exploration, Thunder Bay Regional Research Institute, Thunder Bay, Ontario Canada; 2 Biotechnology Program, Lakehead University, Thunder Bay, Ontario Canada; 3 McDonnell Genome Institute, Washington University School of Medicine, St. Louis, MO USA; 4 NGS platform, Institut Curie, PSL Research University, 26 rue d’Ulm, 75248 Paris, Cedex France; 5 Northern Ontario School of Medicine, Lakehead University, Thunder Bay, Ontario Canada; 6 Department of Biology, Lakehead University, Thunder Bay, Ontario Canada; 7 Department of Chemistry, Lakehead University, Thunder Bay, Ontario Canada; 8 McArdle Laboratory for Cancer Research, University of Wisconsin School of Medicine and Public Health, Madison, WI USA; 9 Institut Curie, PSL Research University, Centre National de la Recherche Scientifique UMR3244, Sorbonne Universités, Paris, France

## Erratum

In the original publication of this article [1], the funding statement omitted the following information, “Work on the NGS platform of the Institut Curie was supported by the Agence Nationale de la Recherche (ANR Investissement d’Avenir) (ANR-10-EQPX-03) and by the France Génomique National infrastructure (ANR-10-INBS-09).”

ᅟ

The final funding statement can be found below:

ᅟ

**Funding**

This work was supported by Natural Sciences and Engineering Research Council of Canada (NSERC) grants to IZ (#355858-2008, #435891-2013, #RGPIN-2015-03855), NSERC Alexander Graham Bell Canada Graduate Scholarship-Doctoral (CGS-D) to RJ (#454402-2014), NSERC Alexander Graham Bell Canada Graduate Scholarship-Masters (CGS-M) to SC (#442618-2013), and an NSERC Undergraduate Student Research Award (USRA) to JB (#483630-2015). Work on the NGS platform of the Institut Curie was supported by the Agence Nationale de la Recherche (ANR Investissement d’Avenir) (ANR-10-EQPX-03) and by the France Génomique National infrastructure (ANR-10-INBS-09). The funding bodies had no role in study design, data collection, data analysis and interpretation, or preparation of the manuscript.
