# Supplementary material for: Functional variants of human papillomavirus type 16 demonstrate host genome integration and transcriptional alterations corresponding to their unique cancer epidemiology
Source: BMC Genomics. 2016 Nov 2;17:851. doi: 10.1186/s12864-016-3203-3 (PMC5094076; doi:10.1186/s12864-016-3203-3)
Supplement: Additional file 1: — Viral and human read tables. Table S1. Viral reads summary. Overall, viral reads make up ~0.0001 to 0.01 % of the total reads, while human reads make up 80 to 85 % of the total reads (the remaining reads are unmapped, to either viral or human sequences). Table S2. Human RefSeq alignment statistics for all samples. NIKS were HPV16 negative organotypic keratinocyte cultures while EPE6 and AAE6 were cultures containing the full genome of HPV16 with either European Prototype E6 or Asian-American E6 variants, respectively. “Aligned” refers to reads overlapping exons, “Exon” refers to reads completely within an exon, and “Exon-Exon” refers to reads overlapping exon junctions. Table S3. Human library size factor for all samples. Library size factors derived from DESeq [52]. (DOCX 15 kb) [file 12864_2016_3203_MOESM1_ESM.docx]

**Table S1. Viral reads summary.** Overall, viral reads make up ~ 0.0001 to 0.01% of the total reads, while human reads make up 80 to 85% of the total reads (the remaining reads are unmapped, to either viral or human sequences).

| **Sample** | **Viral Reads** | **Total Reads** | **%** |
| --- | --- | --- | --- |
| EPE6-1 | 66 | 46.029 x 10^6^ | 0.00014 |
| EPE6-2 | 2014 | 37.344 x 10^6^ | 0.00539 |
| EPE6-3 | 117 | 36.182 x 10^6^ | 0.00032 |
| AAE6-1 | 4505 | 37.518 x 10^6^ | 0.01201 |
| AAE6-2 | 4029 | 39.224 x 10^6^ | 0.01027 |
| AAE6-3 | 4510 | 42.050 x 10^6^ | 0.01073 |

**Table S2. Human RefSeq alignment statistics for all samples.** NIKS were HPV16-negative organotypic keratinocyte cultures while EPE6 and AAE6 were cultures containing the European Prototype HPV16 E6 or Asian-American HPV16 E6 variant cultures, respectively. **“**Aligned” refers to reads overlapping exons, “Exon” refers to reads completely within an exon, and “Exon-Exon” refers to reads overlapping exon junctions.

| **Sample** | **Reads (x10^6^)** | **Aligned (x10^6^)** | **Exon (x10^6^)** | **Exon-Exon (x10^6^)** | **Aligned (%)** | **Exon (%)** | **Exon-Exon (%)** |
| --- | --- | --- | --- | --- | --- | --- | --- |
| NIKS-1 | 41.438 | 33.133 | 16.198 | 16.935 | 79.958 | 48.888 | 51.112 |
| NIKS-2 | 41.729 | 33.103 | 15.930 | 17.172 | 79.328 | 48.124 | 51.876 |
| NIKS-3 | 42.202 | 33.082 | 15.943 | 17.140 | 78.390 | 48.191 | 51.809 |
| EPE6-1 | 46.029 | 36.888 | 17.940 | 18.948 | 80.143 | 48.634 | 51.366 |
| EPE6-2 | 37.344 | 30.845 | 15.137 | 15.708 | 82.596 | 49.074 | 50.926 |
| EPE6-3 | 36.182 | 30.983 | 15.348 | 15.635 | 85.630 | 49.538 | 50.462 |
| AAE6-1 | 37.518 | 31.803 | 15.400 | 16.403 | 84.769 | 48.423 | 51.577 |
| AAE6-2 | 39.224 | 33.213 | 16.117 | 17.096 | 84.674 | 48.527 | 51.473 |
| AAE6-3 | 42.050 | 35.122 | 17.114 | 18.008 | 83.525 | 48.728 | 51.272 |

**Table S3. Human library size factor for all samples.** Library size factors derived from DESeq [1].

| **Sample** | **Library Size Factor** |
| --- | --- |
| NIKS-1 | 0.9913870 |
| NIKS-2 | 0.9666649 |
| NIKS-3 | 0.9699978 |
| EPE6-1 | 1.0824037 |
| EPE6-2 | 0.9072066 |
| EPE6-3 | 0.8962464 |
| AAE6-1 | 1.0532805 |
| AAE6-2 | 1.1016102 |
| AAE6-3 | 1.1151592 |

**SUPPLEMENTAL REFERENCES**

1. Anders S, Huber W. Differential expression analysis for sequence count data. Genome Biol. 2010;11:R106.
