# Supplementary material for: Functional variants of human papillomavirus type 16 demonstrate host genome integration and transcriptional alterations corresponding to their unique cancer epidemiology
Source: BMC Genomics. 2016 Nov 2;17:851. doi: 10.1186/s12864-016-3203-3 (PMC5094076; doi:10.1186/s12864-016-3203-3)

**Figure S1. Plot of normalized mean counts versus log_2_ fold change for the contrast NIKS versus EPE6.**


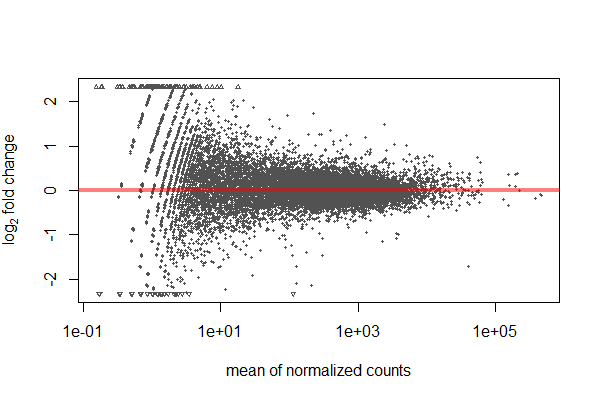


**Figure S2. Plot of normalized mean counts versus log_2_ fold change for the contrast NIKS versus AAE6.**


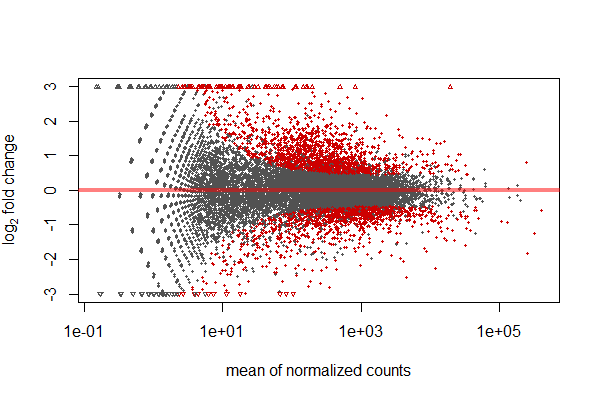


**Figure S3. Plot of normalized mean counts versus log_2_ fold change for the contrast EPE6 versus AAE6.**


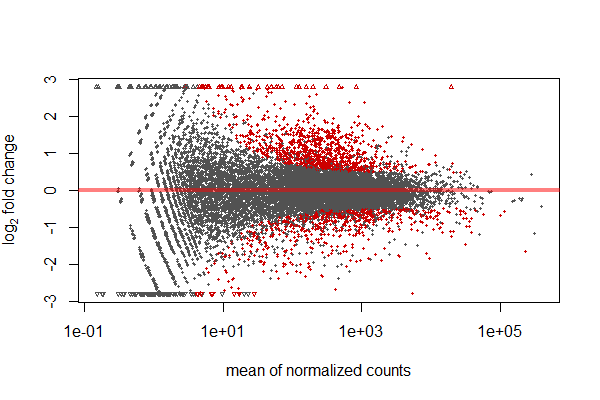


**Figure S4. Empirical and fitted dispersion values plotted against the mean of the normalized human gene-level counts.**


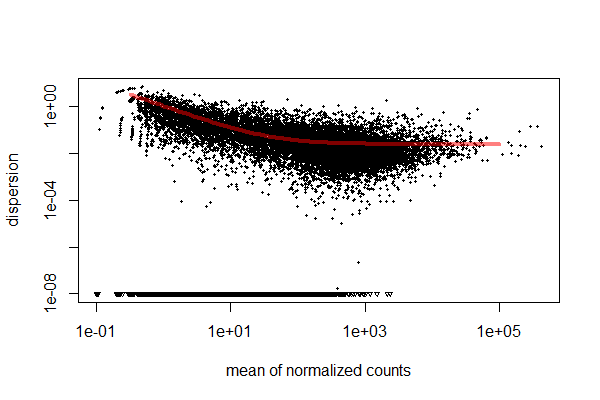


**Figure S5. Heatmap of Euclidean distances between human gene-level counts of samples.** Heatmap and clustering was performed after DESeq variance-stabilizing transformation of human gene-level count data.


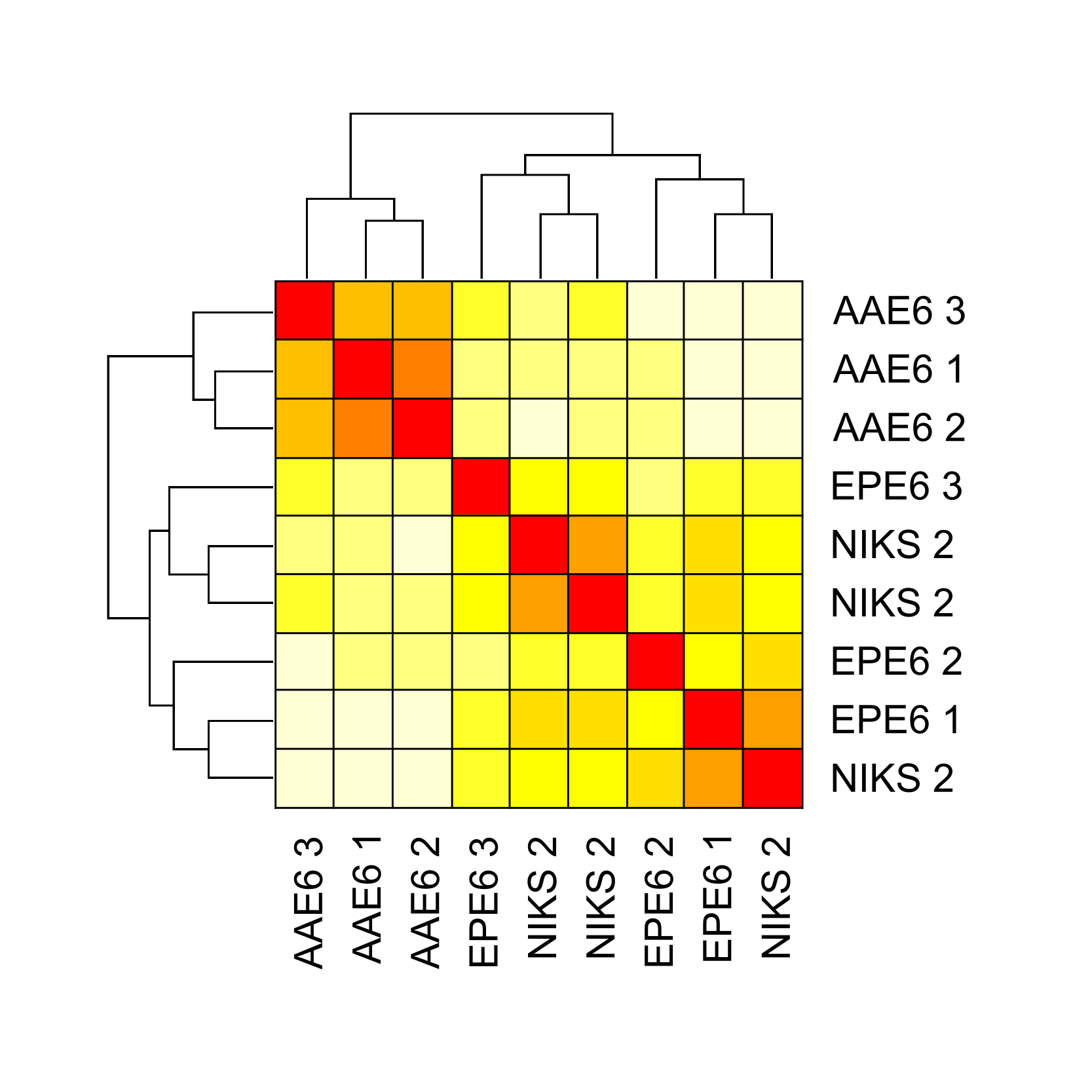

Supplement: Additional file 2: — DESeq plots. Figure S1. Plot of normalized mean counts versus log2 fold change for the contrast NIKS versus EPE6. Red points represent genes that have significant differential expression between the two conditions (false-discovery rate of 10 %, adjusted P < 0.1). No genes were significantly differentially expression between NIKS and EPE6. Figure S2. Plot of normalized mean counts versus log2 fold change for the contrast NIKS versus AAE6. Red points represent genes that have significant differential expression between the two conditions (false-discovery rate of 10 %, adjusted P < 0.1). In total, 3006 genes were significantly differentially expression between NIKS and EPE6. Figure S3. Plot of normalized mean counts versus log2 fold change for the contrast EPE6 versus AAE6. Red points represent genes that have significant differential expression between the two conditions (false-discovery rate of 10 %, adjusted P < 0.1). In total, 1666 genes were significantly differentially expressed between NIKS and EPE6. Figure S4. Empirical and fitted dispersion values plotted against the mean of the normalized human gene-level counts. Red line represents fitted dispersion over the empirical values (black dots). Figure S5. Heatmap of Euclidean distances between human gene-level counts of samples. Heatmap and clustering was performed after DESeq variance-stabilizing transformation of human gene-level count data. (DOCX 204 kb) [file 12864_2016_3203_MOESM2_ESM.docx]
