# Supplementary material for: Functional variants of human papillomavirus type 16 demonstrate host genome integration and transcriptional alterations corresponding to their unique cancer epidemiology
Source: BMC Genomics. 2016 Nov 2;17:851. doi: 10.1186/s12864-016-3203-3 (PMC5094076; doi:10.1186/s12864-016-3203-3)
Supplement: Additional file 4: — Follow-up discussion of host expression analysis. Additional discussion of differential gene expression analysis, pathway-level enrichment, and co-expression networks. Tables S4-S7. top-ten most significant down- or up-regulated genes in AAE6 compared to NIKS or EPE6. (DOCX 30 kb) [file 12864_2016_3203_MOESM4_ESM.docx]

**Additional file 4**

From the top-ten most significant down-regulated genes in AAE6 compared to NIKS (Table S4), CYFIP2 encodes a known p53 target [1] and may be suppressed to prevent apoptosis, or perhaps even be down-regulated as a result of integration as it is located on chromosome 5q33.3. Kelley et al., 2005 [2] noted CYFIP2 as significantly altered by siRNA against E6 or E6AP in HeLa and CaSki cells, likely due to p53 inactivation. A potential biomarker for cervical cancer, AJAP1, has been previously found to be silenced by methylation and implicated in beta-catenin signaling [3]. The transcription factor POU2F3 regulates keratinocyte differentiation and proliferation, encodes a candidate tumour suppressor protein, with silencing by promoter methylation suspected to play a role in cervical cancer [4]. Of the top up-regulated genes in AAE6 compared to NIKS (Table S5), both SLC26A2 and CSF1R are likely increased as a consequence of viral integration into chromosome 5. Interferon-induced IFITM1 is down-regulated in our AAE6 epithelia, which is consistent with a previous report that assessed gene expression in HPV-infected head and neck cancers [5]. The detection of down-regulated RMI2, encoding for a protein involved in homologous recombination [6], could be related to increased genome instability (a possible mechanism for integration). EPE6 to AAE6 had 1,666 significant differentially expressed genes (Additional file 2: Figure S3, Additional file 3 for list of differentially expressed genes between EPE6 and AAE6). Of these genes, 666 were down-regulated while 1,000 were up-regulated in AAE6 compared to EPE6. The top-ten down-regulated genes in AAE6 compared to EPE6 are presented in Table S6 and include 6 of the same genes as found in the AAE6 to NIKS comparison. The top-ten significant up-regulated genes in AAE6 compared to EPE6 are presented in Table S7 and include 8 of the same genes as found in the AAE6 to NIKS comparison.

To increase the stringency of differentially expressed genes we separated down-regulated and up-regulated genes from each pairwise comparison below an adjusted *P*-value threshold of 10^-5^. For genes differentially expressed in AAE6 compared to NIKS, the stringent cut-off yielded 61 down-regulated genes (61/1,312 = 4.65% highly significant down-regulated genes) and 248 up-regulated genes (248/1,694 = 14.64% highly significant up-regulated genes). For genes differentially expressed in AAE6 compared to EPE6, the stringent cut-off yielded 26 down-regulated genes (26/666 = 3.90% highly significant down-regulated genes) and 152 up-regulated genes (152/1,000 = 15.20% highly significant up-regulated genes). With only highly significant differentially expressed genes identified, we then proceeded to pathway-level analysis to determine which host biological processes were enriched given down-regulated and up-regulated sets of genes.

Enrichment of host biological processes within the highly significant sub-sets of differentially expressed human genes was determined using the Gene Ontology (GO) Term Enrichment Service hosted on the AmiGO 2 website [7]. Terms were considered significantly enriched if the Bonferroni-corrected *P*-value was less than 0.05. In total, 50 GO terms were significantly enriched among highly significant down-regulated genes in AAE6 compared to NIKS (Figure 5A for top-ten, Additional file 5 for full list). Enrichment of these biological processes among highly significant down-regulated genes reflects the poor differentiation and tumourigenic tissue phenotype caused by AAE6 [8], now evidenced by transcriptome-level data. Additionally, 176 GO terms were significantly enriched among highly-significant up-regulated genes (Figure 5B for top-ten, Additional file 5 for full list), reflecting the proliferative phenotype caused by AAE6 [8] and providing further evidence for variant-specific transcriptome-wide changes.

When comparing AAE6 to EPE6, only 4 GO terms were significantly enriched among highly significant down-regulated genes (Figure 6A, Additional file 5 for full list). Enrichment of these lipid metabolism biological processes among highly significant down-regulated genes is a finding that sheds new light on HPV-centered host-pathogen interactions and HPV-driven tumourigenesis. Notable down-regulated genes were ALDH1A1/A2. These aldehyde dehydrogenases catalyze the synthesis of retinoic acid which interestingly suppresses viral oncogene expression [9]. A decrease of their expression could perhaps be permitting, at least in part, to the continued over-expression of oncogenes by AAE6. For highly significant up-regulated genes in AAE6 compared to EPE6, 231 GO terms were significantly enriched (Figure 6B for top-ten, Additional file 5 for full list). Enrichment of cell cycle and proliferation biological processes was confirmed in AAE6 over EPE6, further demonstrating AAE6’s enhanced tumourigenic potential over EPE6 [8], thought to be due in part to an enhanced Warburg effect (Cuninghame et al, in preparation: unpublished observations). Pathway-level analysis revealed that many of the changes due to AAE6 were related to increased cell cycle and DNA synthesis, which are commonly promoted pathways in tumourigenesis [10, 11], and more specifically, viral tumourigenesis [12]. In the synthesis of DNA pathway, for example, nearly every gene is up-regulated, with the exception of lower CDKN1A (coding for p21). This potent cyclin-dependent kinase inhibitor is functionally regulated by p53, with its down-regulation associated with invasive cervical cancer [13].

In addition to pathway-level analysis, Cytoscape was used for visualization of co-expressed genes from the highly significant down- and up-regulated genes in AAE6 compared to EPE6. Visualizations and interpretations with networks, including co-expression networks, can play an important role in analyzing the large amount of data generated from high-throughput experiments [14] since multiple levels of information can be presented simultaneously (such as the degree of gene co-expression, fold change, and functional annotations). The co-expression network for down-regulated genes reveals four distinct clusters of genes (Figure 7A). The strongest correlation in the first cluster was between KIAA0040 (an uncharacterized protein) and LIPG (lipase, endothelial), while WDR63 (WD repeat domain 63) was the most down-regulated due to AAE6. In the second cluster, the strongest correlation was between CYP4F22 (cytochrome P450, family 4, subfamily F, polypeptide 22) and KRT6B (keratin 6B). CYP4F22, a cytochrome P450, is likely involved in keratinocyte differentiation [15], so it is not surprising that it is strongly correlated to a cytokeratin. Mutations of this cytochrome gene lead to the skin disorder ichthyosis (scaly skin) [16], which further supports it has a role in keratinocyte differentiation. The most down-regulated gene in this second cluster was AJAP1, discussed above as a methylated gene in cervical cancer. In the third down-regulated cluster the strongest correlation was between INPP5D (inositol polyphosphate-5-phosphatase) and LOC375010 (ankyrin repeat domain 20 family, member A pseudogene). INPP5D is a negative regulator of the PI3K (phosphoinositide 3-kinase) pathway [17], a common pathway involved in proliferation and tumourigenesis, so INPP5D’s down-regulation coincides with the tumourigenic potential of AAE6. The most down-regulated gene in this cluster was DAPK1 (death-associated protein kinase 1), which is another gene that is known to have its promoter methylated in cervical cancer [18]. The fourth cluster of down-regulated genes contained only two genes: ANTXR2 (anthrax toxin receptor 2) and CSMD3 (CUB and sushi multiple domains). ANTXR2 binds to extracellular matrix (ECM) proteins collagen IV and laminin, which suggests it may have a role in ECM adhesion in mouse studies [19]. Interestingly, CSMD3 has been associated with the fragile site FRA8C at chromosome 8q24 in cervical carcinoma with HPV integration [20].

Looking at the highly significant and co-expressed up-regulated genes, five distinct clusters were observed (Figure 7B). The first cluster was significantly enriched for “DNA replication” (GO:0006260) due to the high number of up-regulated and strongly co-expressed gene such as the MCM’s. The most up-regulated gene was SLC26A2, which we previously discussed as up-regulated as a result of viral integration. The strongest correlations were between MCM7 and MCM3, as well as DSN1 and CHEK1. The second cluster was significantly enriched for “macrophage colony-stimulating factor signaling pathway” (GO:0038145) due to the presence of CSF1R, likely upregulated on chromosome 5 due to viral integration (as discussed above). This gene was strongly co-expressed with TCAM1P (testicular cell adhesion molecule 1, pseudogene). The remaining three clusters were significantly enriched for cellular division processes. Enrichment and up-regulation of these biological processes is consistent with the proliferative and tumourigenic phenotype in AAE6.

**Table S4.** Top-ten most significant down-regulated genes in AAE6 compared to NIKS.

| **Gene Symbol** | **Gene Name** | **Fold**  **Change** | **Adjusted**  ***P*-value** |
| --- | --- | --- | --- |
| CYFIP2 | cytoplasmic FMR1 interacting protein 2 | 0.15 | 6.92 x 10^-21^ |
| AJAP1 | adherens junctions associated protein 1 | 0.14 | 7.37 x 10^-19^ |
| CSMD3 | CUB and Sushi multiple domains 3 | 0.21 | 4.19 x 10^-15^ |
| ANTXR2 | anthrax toxin receptor 2 | 0.31 | 1.74 x 10^-12^ |
| EFNB3 | ephrin-B3 | 0.22 | 2.46 x 10^-12^ |
| SDK2 | sidekick cell adhesion molecule 2 | 0.16 | 5.97 x 10^-12^ |
| SMTN | smoothelin | 0.35 | 2.92 x 10^-11^ |
| EDA2R | ectodysplasin A2 receptor | 0.19 | 2.94 x 10^-11^ |
| POU2F3 | POU class 2 homeobox 3 | 0.28 | 1.84 x 10^-10^ |
| FLRT2 | fibronectin leucine rich transmembrane protein 2 | 0.38 | 3.74 x 10^-10^ |

**Table S5.** Top-ten most significant up-regulated genes in AAE6 compared to NIKS.

| **Gene Symbol** | **Gene Name** | **Fold**  **Change** | **Adjusted**  ***P*-value** |
| --- | --- | --- | --- |
| SLC26A2 | solute carrier family 26 (anion exchanger), member 2 | 114.19 | 2.14 x 10^-173^ |
| CSF1R | colony stimulating factor 1 receptor | 407.82 | 4.70 x 10^-112^ |
| GPAT2 | glycerol-3-phosphate acyltransferase 2, mitochondrial | 387.18 | 4.76 x 10^-80^ |
| IFITM1 | interferon induced transmembrane protein 1 | 9.98 | 7.56 x 10^-45^ |
| MCM2 | minichromosome maintenance complex component 2 | 6.25 | 4.95 x 10^-34^ |
| MEST | mesoderm specific transcript | 14.63 | 5.97 x 10^-33^ |
| CDCA7 | cell division cycle associated 7 | 6.40 | 1.21 x 10^-32^ |
| RMI2 | RecQ mediated genome instability 2 | 8.80 | 2.43 x 10^-29^ |
| MCM6 | minichromosome maintenance complex component 6 | 4.68 | 3.23 x 10^-24^ |
| KLHL35 | kelch-like family member 35 | 33.26 | 1.39 x 10^-23^ |

**Table S6.** Top-ten most significant down-regulated genes in AAE6 compared to EPE6.

| **Gene Symbol** | **Gene Name** | **Fold**  **Change** | **Adjusted *P*-value** |
| --- | --- | --- | --- |
| EFNB3 | ephrin-B3 | 0.21 | 3.57 x 10^-13^ |
| AJAP1 | adherens junctions associated protein 1 | 0.20 | 7.32 x 10^-13^ |
| CYFIP2 | cytoplasmic FMR1 interacting protein 2 | 0.24 | 6.01 x 10^-12^ |
| PAQR5 | progestin and adipoQ receptor family member V | 0.32 | 6.59 x 10^-11^ |
| CSMD3 | CUB and Sushi multiple domains 3 | 0.26 | 7.60 x 10^-11^ |
| DAPK1 | death-associated protein kinase 1 | 0.24 | 1.02 x 10^-10^ |
| ANTXR2 | anthrax toxin receptor 2 | 0.36 | 9.30 x 10^-10^ |
| SMTN | smoothelin | 0.40 | 5.63 x 10^-09^ |
| LOC100216001 | long intergenic non-protein coding RNA 704 | 0.22 | 9.07 x 10^-08^ |
| MFAP5 | microfibrillar associated protein 5 | 0.35 | 1.40 x 10^-07^ |

**Table S7.** Top-ten most significant up-regulated genes in AAE6 compared to EPE6.

| **Gene Symbol** | **Gene Name** | **Fold**  **Change** | **Adjusted *P*-value** |
| --- | --- | --- | --- |
| SLC26A2 | solute carrier family 26 (anion exchanger), member 2 | 118.42 | 1.98 x 10^-173^ |
| CSF1R | colony stimulating factor 1 receptor | 551.09 | 1.28 x 10^-113^ |
| GPAT2 | glycerol-3-phosphate acyltransferase 2, mitochondrial | 290.62 | 6.59 x 10^-78^ |
| IFITM1 | interferon induced transmembrane protein 1 | 6.95 | 7.74 x 10^-34^ |
| MCM2 | minichromosome maintenance complex component 2 | 5.20 | 4.83 x 10^-28^ |
| CDCA7 | cell division cycle associated 7 | 5.25 | 2.40 x 10^-26^ |
| RMI2 | RecQ mediated genome instability 2 | 5.84 | 1.18 x 10^-20^ |
| MEST | mesoderm specific transcript | 7.06 | 1.19 x 10^-20^ |
| LOC254559 | long intergenic non-protein coding RNA 925 | 12.34 | 2.50 x 10^-20^ |
| C1R | complement component 1, r subcomponent | 7.27 | 1.76 x 10^-19^ |

**SUPPLEMENTAL REFERENCES**

1. Jackson II RS, Cho YJ, Stein S, Liang P. CYFIP2, a Direct p53 Target, is Leptomycin-B Sensitive. Cell Cycle. 2007;6:95-103.
2. Kelley ML, Keiger KE, Lee CJ, Huibregtse JM. The global transcriptional effects of the human papillomavirus E6 protein in cervical carcinoma cell lines are mediated by the E6AP ubiquitin ligase. J Virol. 2005;79:3737-47.
3. Chen YC, Huang RL, Huang YK, Liao YP, Su PH, Wang HC, et al. Methylomics analysis identifies epigenetically silenced genes and implies an activation of β‐catenin signaling in cervical cancer. Int J Cancer. 2014;135:117-27.
4. Zhang Z, Huettner PC, Nguyen L, Bidder M, Funk MC, Li J, et al. Aberrant promoter methylation and silencing of the POU2F3 gene in cervical cancer. Oncogene. 2006;25:5436-45.
5. Schlecht NF, Burk RD, Adrien L, Dunne A, Kawachi N, Sarta C, et al. Gene expression profiles in HPV‐infected head and neck cancer. J Pathol. 2007;213:283-93.
6. Deans AJ, West SC. DNA interstrand crosslink repair and cancer. Nat Rev Cancer. 2011;11: 467-80.
7. Carbon S, Ireland A, Mungall CJ, Shu S, Marshall B, Lewis S. AmiGO: online access to ontology and annotation data. Bioinformatics. 2009;25:288-9.
8. Jackson R, Togtema M, Lambert PF, Zehbe I. Tumourigenesis Driven by the Human Papillomavirus Type 16 Asian-American E6 Variant in a Three-Dimensional Keratinocyte Model. PLOS ONE. 2014;9:e101540.
9. Faluhelyi Z, Rodler I, Csejtey A, Tyring SK, Ember IA, Arany I. All-trans retinoic acid (ATRA) suppresses transcription of human papillomavirus type 16 (HPV16) in a dose-dependent manner. Anticancer Res. 2004;24:807-9.
10. Hanahan D, Weinberg RA. The hallmarks of cancer. Cell. 2000;100:57-70.
11. Hanahan D, Weinberg RA. Hallmarks of cancer: the next generation. Cell. 2011;144:646-74.
12. Mesri EA, Feitelson MA, Munger K. Human Viral Oncogenesis: A Cancer Hallmarks Analysis. Cell Host Microbe. 2014;15:266-82.
13. Bahnassy AA, Zekri AR, Alam El-Din HM, Aboubakr AA, Kamel K, El-Sabah MT, et al. The role of cyclins and cyclins inhibitors in the multistep process of HPV-associated cervical carcinoma. J Egypt Natl Canc Inst. 2006;18:292-302.
14. Merico D, Gfeller D, Bader GD. How to visually interpret biological data using networks. Nat Biotech. 2009;27:921-4.
15. Sasaki K, Akiyama M, Yanagi T, Sakai K, Miyamura Y, Sato M, et al. CYP4F22 is highly expressed at the site and timing of onset of keratinization during skin development. J Dermatol Sci. 2012;65:156-8.
16. Fischer J. Autosomal recessive congenital ichthyosis. J Invest Dermatol. 2009;129:1319-21.
17. Huang X, Shen Y, Liu M, Bi C, Jiang C, Iqbal J, et al. Quantitative Proteomics Reveals that miR-155 Regulates the PI3K-AKT Pathway in Diffuse Large B-Cell Lymphoma. Am J Pathol. 2012;181:26-33.
18. Banzai C, Nishino K, Quan J, Yoshihara K, Sekine M, Yahata T, et al. Promoter methylation of DAPK1, FHIT, MGMT, and CDKN2A genes in cervical carcinoma. Int J Clin Oncol. 2014;19:127-32.
19. Reeves C, Charles-Horvath P, Kitajewski J. Studies in Mice Reveal a Role for Anthrax Toxin Receptors in Matrix Metalloproteinase Function and Extracellular Matrix Homeostasis. Toxins. 2013;5:315-26.
20. Ferber MJ, Eilers P, Schuuring E, Fenton JA, Fleuren GJ, Kenter G, et al. Positioning of cervical carcinoma and Burkitt lymphoma translocation breakpoints with respect to the human papillomavirus integration cluster in FRA8C at 8q24. 13. Cancer Genet Cytogenet. 2004;154:1-9.
